# Wear Characterization of Phenol-Formaldehyde-Based Saguvani Wood–Polymer Composite—An ANOVA Approach

**DOI:** 10.3390/ma16144999

**Published:** 2023-07-14

**Authors:** B. T. Ramesh, R. S. Ramesh, Javed Sayyad, Arunkumar Bongale

**Affiliations:** 1 Department of Robotics and Automation Engineering, Symbiosis Institute of Technology (SIT), Symbiosis International (Deemed University) (SIU), Lavale, Pune 412115, Maharashtra, India; jksayyad23@gmail.com (J.S.); arunbongale1980@gmail.com (A.B.); 2 Jain Institute of Technology, Davangere 577003, Karnataka, India; rsrrampura@gmail.com

**Keywords:** wear, phenol formaldehyde, ANOVA, wood–polymer composite, rotary-drum-type blender

## Abstract

In this part of the research work, the Taguchi approach is used to analyze the weight wear loss of PF-based 10% chemically treated saguvani wood–polymer composite under dry sliding conditions. The fabrication of PF-based wood–polymer composite consisting of 10% chemically treated saguvani wood particles as reinforcement material filled with coconut shell powder is used. The rotary-drum-type blender is used for uniform mixing of reinforcement materials with resin as per the calculated volume ratio. The inclusion of coconut shell powder as secondary particles in the PF-based wood plastic composite minimizes the wearability of the composite. The Taguchi method is used successfully to analyze the wear behavior of the PF-based wood–polymer composite with sliding speed, load, and sliding distance as control parameters. The experimental work reveals that the composite C1 shows minimum wear loss compared to the other composite specimens, C2 and C3. And the most influential parameter that causes more wear is the sliding distance among the three control parameters.

## 1. Introduction

Wear is one of the important mechanical properties that has to be investigated for all the materials which are used for structural applications [1,2]. And also in most material usage, a low wear rate is desirable. Particularly, in the case of wood–polymer composites, as they have been used as decking, railing, automotive, and insulating materials, they should have wear-resistance properties. Comprehension of the efficacy and longevity of composite materials in real-world applications requires a thorough comprehension of their wear characteristics. Using an ANOVA (analysis of variance) approach, we investigate the wear properties of phenol-formaldehyde (PF)-based saguvani wood–polymer composite (SWPC). In particular, SWPC has drawn significant interest as a sustainable alternative to solid timber in a variety of industries, including the construction, furniture, and automotive industries. By coupling wood fibers or particulates with a polymer matrix, these composites exhibit desirable characteristics, including high strength, dimensional stability, and decay resistance. The selection of a polymer matrix has a substantial impact on the overall efficacy of wood–polymer composites. The thermosetting polymer phenol formaldehyde (PF) is well-known for its superior mechanical strength, chemical resistance, and thermal stability. When combined with saguvani wood, which is renowned for its durability and dimensional stability, PF-based saguvani wood–polymer composite demonstrates promise for wear-resistant applications.

Ashutosh Pattanaik et al. [3] reported the wear behavior of epoxy resin polymer composite fabricated by using the ultrasonic stirring method. The eroded particles which are present between the surface of the plate and the sample causes secondary wear. The inclusion of boron in a polymer composite substantially enhances the wear resistance of composites [4]. The mineral additives were added to mixtures in different weight ratios as 0 to 66% by replacing the resin. The wear increases slightly with increasing filler content by up to 33% because of poor interfacial bonding between the resin and reinforcement material. The factors sliding distance, load, and time significantly influence the wear rate of composites [5]. The wear resistance of graphite micro-particle-filled polyamide composite specimens was improved by using graphite fillers. The best possible wear resistance ability was obtained at the filler inclusion of 25% weight fraction [6]. High specific strength, wear resistance, and the specific modulus of polymer composites make them suitable for structural, aerospace, and automobile applications [7]. S.P. Thorat et al. [8] observed in their work that the wear resistance and hardness of polymeric composite prepared by PTFE improved by adding filler materials such as carbon, graphite, and glass fibers while the coefficient of friction was slightly affected and remained low for the fabricated composite samples.

In this research, the wear performance of PF-based saguvani wood–polymer composite is analyzed using ANOVA. ANOVA permits the comparison of multiple groups or conditions, in this instance, various formulations of composite materials or processing parameters. By conducting a systematic analysis, we hope to determine if there are significant differences between the various composite formulations in terms of wear loss. The ANOVA method provides a statistical framework for evaluating the impact of various factors on wear performance, such as the composition of the composite, test conditions for wear, and other pertinent variables. By quantifying the variability and significance of these factors, we can gain insight into the key parameters that influence wear resistance and optimize the formulation of the composite material accordingly. This study seeks to contribute to a comprehension of the wear behavior of PF-based saguvani wood–polymer composites. By employing an ANOVA method, we hope to identify the significant factors influencing wear performance, which will aid in the development of enhanced composite materials for applications involving extensive wear. The Taguchi method is used successfully to analyze the wear behavior of PF-based wood–polymer composite with sliding speed, load, and sliding distance as control parameters. The experimental work reveals that the composite C1 shows minimum wear loss compared to other composite specimens C2 and C3. And the most influential parameter that causes more wear is the sliding distance among the three control parameters.

Sunil Thakur and S.R. Chauhan [9] have investigated the tensile, flexural, compressive, and wear properties of cenosphere-filled vinylester composites. The micro-sized cenosphere used as fillers shows significant wear resistance. The abrasive wear behavior of glass-filled epoxy composites was reported by Sudarshan Rao K et al. [10]. They have observed that one of the parameters, the load has a substantial impact on the wear of the composite. Jian et al. in 2022 reviewed the flexural properties of wood–plastic composites. The wear resistance of glass vinylester composite has been significantly improved by the addition of fly ash filler material [11]. The hardness and wear resistance of the PTFE composite can be improved by adding filler materials such as carbon, graphite, and glass fibers. The addition of these materials is so effective in impeding large-scale fragmentation of PTFE and minimizes the wear rate [12]. Antaryami Mishra [13] studied the wear behavior of epoxy composite filled with teak wood sawdust with a varying weight fraction of teak wood as 10, 15, and 20, respectively. The wood particle sizes of 150 µm, 212 µm, and 300 µm were used in the study. The wood particle size of 300 µm shows minimum wear compared with the 150 µm and 212 µm size particles in the epoxy composite.

The contacting bodies’ rising temperatures reduce the flow stresses of the materials to some amount, which causes the size of the plastic zone in the contacting bodies’ subsurface to develop. When the usual load is above a specific level, the friction coefficient and wear rate rise with higher sliding speed [14,15,16]. The objective of this work is to study the wear behavior of phenol formaldehyde-based saguvani (10% chemically treated) wood–plastic composite and hence to predict the impact of factors like sliding speed, load, and sliding distance. To improve the compatibility between wood and polymers, chemical treatment was selected. The selection of 10% chemical treatment was based on prior research, where it has been found to yield desirable properties and to achieve the desired balance between surface modification and processing difficulties. The materials and methods used for the wear characterization are presented under materials and methods. The Taguchi method is used to optimize the parameters which have been considered in this experimentation.

## 2. Materials and Methods

**Figure 1:**
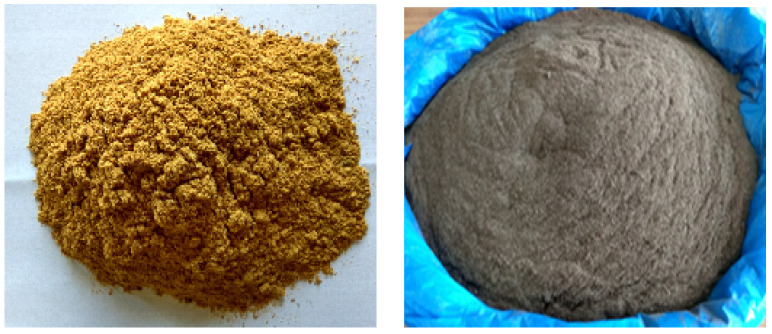
Saguvani and Coconut shell powder.

Wood waste generated from wood products, such as sawdust, offers excellent potential for fabricating composite materials. Sawdust is a by-product of various wood processing operations and is readily available in the environment. Rather than being disposed of as waste, sawdust can be effectively utilized in producing composite materials [17]. This work uses saguvani wood waste and coconut shell powder as reinforcement. Wood debris from saguvani was obtained from a nearby sawmill in Davangere, Karnataka. Meanwhile, a local supplier in Hassan, Karnataka, provided coconut shell powder that was chosen for its outstanding modulus and high strength qualities. Notably, agricultural waste is used to create coconut shell powder. These components are thoroughly processed before being added to the composite samples to remove contamination and avoid adverse effects during production. Figure 1 shows the saguvani and coconut shell powder used in this work. The saguvani wood waste collected from local sawmills was thoroughly screened to remove any impurities present in them. The wood flour of 850 µm was selected as reinforcement material, and PF was selected as matrix material. Coconut shell powder was used as secondary reinforcement material. These selected wood wastes were subjected to 10% chemical treatment to modify the surface of the wood material. The composite specimens were fabricated according to the calculated volume fraction by using the hot press method. The test specimens of circular 10 mm diameter and 20 mm length were prepared and tested as per the ASTM E 1530.

Phenol formaldehyde (PF) is blended as the retort of part phenol with part formaldehyde. The distinctive properties like the ease of molding, excellent chemical resistance, and good weather resistance make it useful as a matrix material. AKOLITE Synthetic Resins, Mangalore, Karnataka procures phenol formaldehyde (PF) which has been used as a matrix material in this work. Table 1 shows the density and grain fineness number of reinforcement materials used in this work as these values will help in the depiction of composite materials. The formulation of composites are as shown in Table 2. SWPC10T30 determines saguvani wood polymer composites (SWPC) 10% chemically treated 30% wood composition.

Table 3 tabulates the grade and brand of all chemicals used in this study, which shows sodium hydroxide, benzyl chloride, and acrylic acid used to modify the surface of the wood flour.

**Figure 2:**
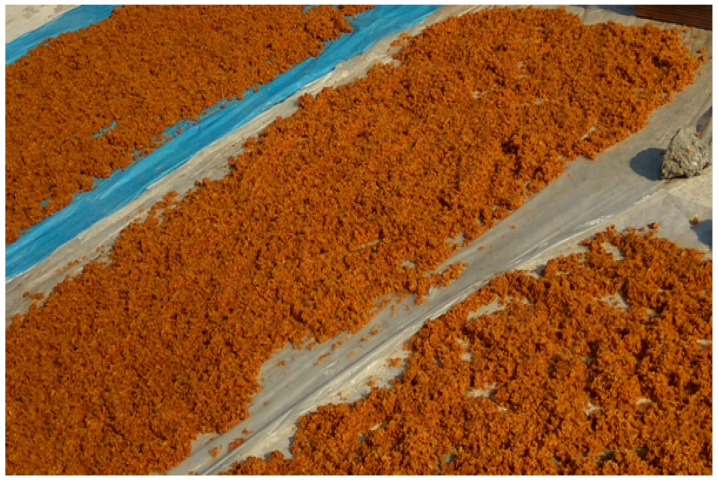
Saguvani wood flour after 10% chemical treatment.

The saguvani wood flour and 850 μm sized coconut shell particles first undergo a detailed screening procedure to eliminate impurities. Saguvani wood flour is specifically treated chemically using a 10% solution of sodium hydroxide, benzyl chloride, and acrylic acid. The first step in the treatment involves soaking the wood flour in a 10% sodium hydroxide solution for 30 min. The treated flour is rinsed with distilled water to remove any excess alkali. The dried wood flour is then dehydrated for twenty-four h at 100 °C in an oven. The wood flour treated with sodium hydroxide is then soaked for 15 min in a benzyl chloride solution. Following this procedure, the flour is once again cleaned with distilled water before being dried for a further twenty-four h at 100 °C. The wood flour is then subjected to a 30 min chemical curing procedure using acrylic acid at 50 °C. The flour is then washed once more to eliminate any leftover moisture or water. The saguvani wood flour is manufactured and processed to satisfy the necessary quality criteria for the composite samples by undergoing these meticulously regulated chemical processes. The reinforcement materials at 100 °C for twenty-four h are maintained in a hot air oven to remove moisture content if any is present in them. According to Figure 2, the chemically treated saguvani wood flour is utilized in the process. It is thoroughly mixed with phenol formaldehyde in a rotating barrel-type whizzer, following the prescribed volume proportions. This step ensures a complete and uniform combination of the treated wood flour with the phenol formaldehyde, crucial for the subsequent stages of the process.

### Experimental Design

The construction of room-temperature self-healing materials with faster self-healing speed and higher mechanical strength is an area of active research and development in the field of materials science and engineering [18]. Several methods and tests can be employed to evaluate the bonding strength and water resistance of the proposed chemical wood bonding interface [19].

**Figure 3:**
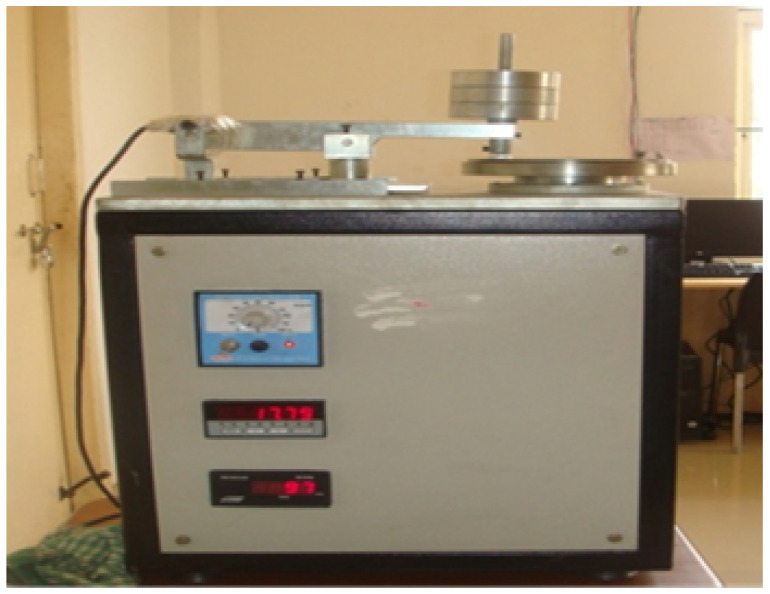
Pin-on-disc wear test machine.

To evaluate the wear performance of PF-based saguvani wood–polymer composite with respect to dry sliding conditions, the wear characterization is performed through pin-on-disc-type wear testing instrument as per ASTM G99-95a standard [15]. Figure 3 shows a pin-on-disc wear testing instrument used for this work. The tests are conducted with a sliding speed of 1.30, 2.61, and 3.92 m/s, a load of 10 N, 20, and 30 N, and a sliding distance of 392 m, 785 m, and 1177 m. Six replicas of fabricated composite test specimens were tested.

A useful analysis tool for modeling and examining the impact of control parameters on cognitive measures is the Taguchi design of the experiment. The crucial step in the design of experiments is the determination of control parameters. The three levels of values for the process parameters are shown in Table 4. The orthogonal L27 (313) design is shown in Table 5 and was used to examine the effects of three control factors.

## 3. Results and Discussion

The experimental data for sliding wear are reported for SWPC10T30 (Composite-C1), SWPC10T40 (Composite-C2), and SWPC10T50 (Composite-C3) composites, and they are analyzed by Minitab Version 17, particularly suitable for design of experiments. When evaluating the experimental data, the interactions between the control variables are taken into account. The average wear loss for each factor at various levels is indicated by the mean response.

### 3.1. ANOVA and Effects of Factors for SWPC

Taguchi’s design of the experiment is a powerful analysis tool for modeling and analysing the influence of control factors on performance output. The selection of control parameters is the vital stage in the design of experiments. Each parameter is set based on the load, speed, and sliding distance calculation. The 10, 20, and 30 N load is selected since saguvani wood material is fibrous and close-grained.

#### 3.1.1. SWPC10T30 (Composite-C1)

It is observed from Table 6 that sliding distance (P = 32.30%) is the key factor as it is responsible for more wear weight loss trailed by the load (P = 9.81%) and sliding speed (P = 3.98%). The sliding distance, load exerts substantial effect on the sliding wear. The relations among speed/load, load/distance, and speed/distance have minimum influence on wear loss. It can be concluded from Table 7 that sliding distance has a major influence on wear loss. The matrix material PF of composite breaks away from the reinforcement material (wood flour) and other additives because of the embrittlement of the matrix as compared to the reinforcement. Due to this reinforcement material comes in contact with the disc and it leads to more wear at the highest sliding distance. Table 8 displays the result of ANOVA for composite C1. The table shows that the sliding distance (P = 53.96%), load (P = 21.53%), sliding speed (P = 12.04%), and the interaction between (A × C) speed and sliding distance (P = 5.37%) have major effects on the wear loss and hence these are more considerable factors for analysis. However, the relations between (A × B) sliding speed and load (P = 3.36%), (B × C) load and sliding distance (P = 0.97%) have less effect on the wear loss.

From response Table 9 for the signal-to-noise ratio (S/N ratio) of composite C1, it is observed that the sliding distance has a key impact on wear loss trailed by (B) load and (A) sliding speed. The increase in load and sliding distance develops heat at the interface between matrix and filler material. The heat developed at the interface leads thermal penetration to occur and hence weakens the bond between them. This in turn maximizes the wear of composite under dry sliding conditions.

**Figure 4:**
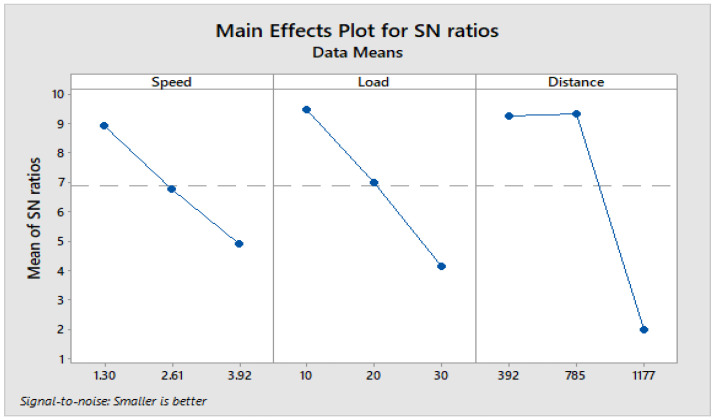
Main effects plot for S/N ratios of Composite-C1.

**Figure 5:**
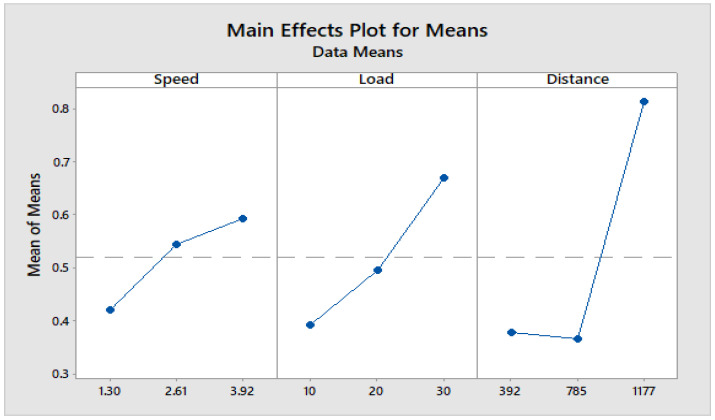
Main effects plot for means of Composite-C1.

**Figure 6:**
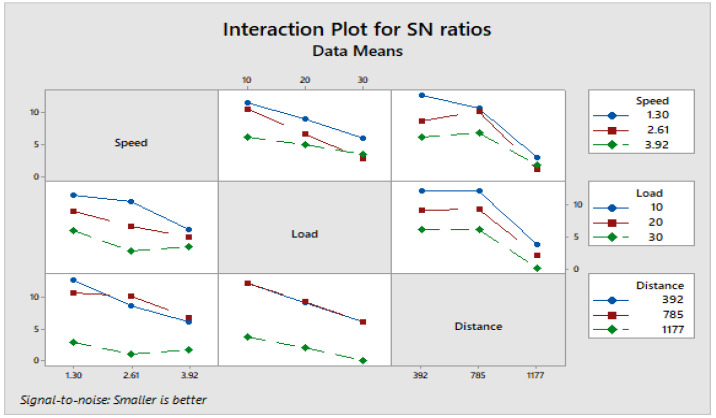
Interaction plot for S/N ratios of Composite-C1.

**Figure 7:**
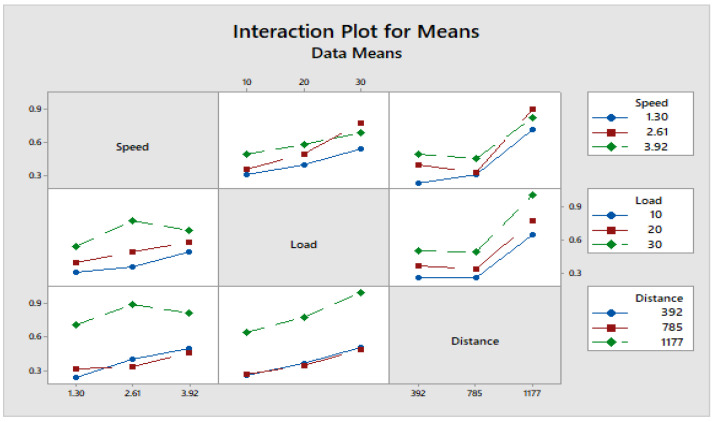
Interaction plot for means of Composite-C1.

Figure 4 depicts how wear loss of the composite specimen C1 is affected by three control parameters. The evaluation of these data reveals that the factor combination of A1, B1, and C2 provides the least amount of wear. As illustrated in Figure 5, the combination of components A3, B3, and C3 results in the least amount of wear loss. It is observed (Figure 6 and Figure 7) that the interaction (B × C) shows a significant effect on the wear loss. There is an appreciable reduction in wear loss of composite C1 with 5% addition of coconut shell powder. As the % of inclusion of coconut shell powder increases, wear will be minimum.

#### 3.1.2. SWPC10T40 (Composite-C2)

The sliding distance (P = 57.67%), sliding speed (P = 16.85%), load (P = 10.24%), and interaction of (B) sliding speed × (C) sliding distance (P = 6.64%) have a significant influence on the wear loss (Table 10). However, interactions of speed × load (A × B) (P = 3.09%) and load × distance (B × C) (P = 1.48%) do not have a substantial influence on wear loss as their values are reasonably less than the error (P = 4.57%) so they are neglected. From the response table for means, Table 11, it is observed that sliding distance has a key influence on wear loss followed by sliding speed and load.

It can be observed (Table 12) that the sliding distance (P = 52.49%), load (P = 11.57%), sliding speed (P = 19.97%), and the interaction between (A × C) speed and sliding distance (P = 7.33%), (A) speed, and (B) load (P = 5.29%) have major stimulus on the wear loss and hence these are statistically significant. However, the interaction between (B × C) load and sliding distance (P = 1.50%) has less influence on the wear loss.

From response Table 13 for the S/N ratio of composite C2, it is observed that the control parameter (C) sliding distance has a major influence on wear loss followed by (A) sliding speed and (B) load. The substantial increase in sliding distance and speed develops the temperature at the interface and hence results in more wear.

**Figure 8:**
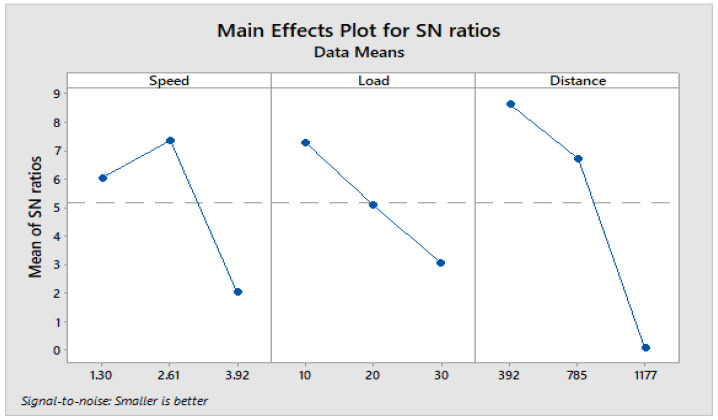
Main effects plot for S/N ratios of Composite-C2.

**Figure 9:**
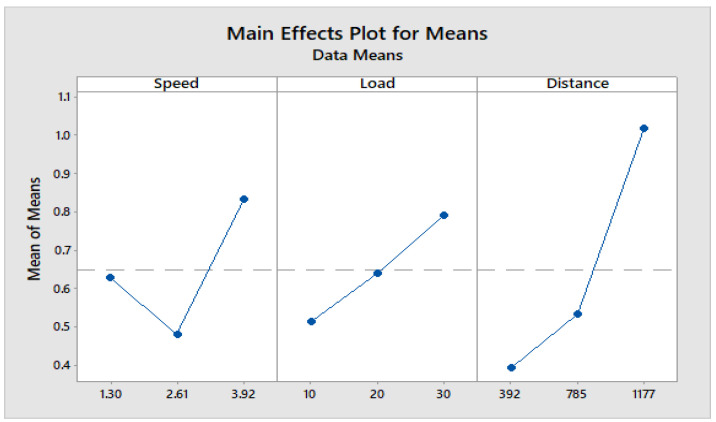
Main effects plot for means of Composite-C2.

**Figure 10:**
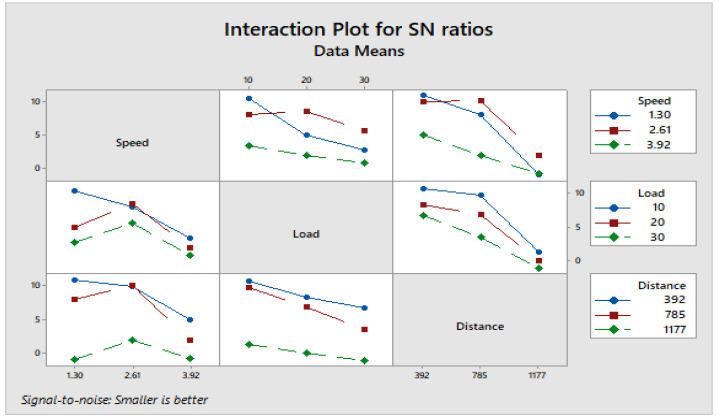
Interaction plot for S/N ratios of Composite-C2.

**Figure 11:**
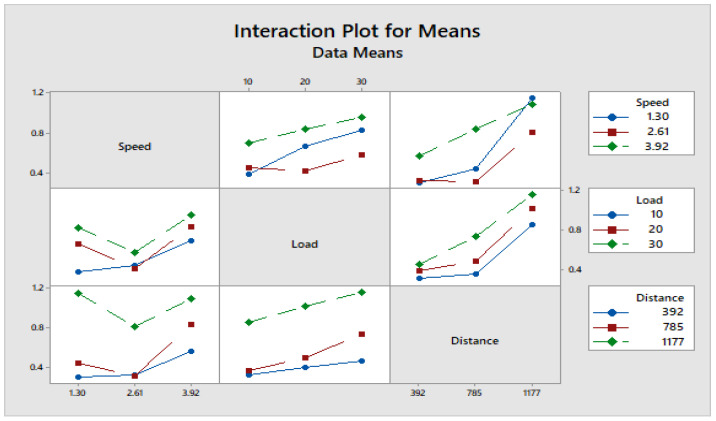
Interaction plot for means of Composite-C2.

Figure 8 shows the influence of three control factors on weight wear loss of composite specimens C2. The main effects plot for means of Composite-C2 is shown in Figure 9, and it can be drawn from the figure that factors A3, B3, and C3 exhibit the minimum wear rate. The interaction graphs are shown in Figure 10 and Figure 11. From these figures, it is observed that the interaction (B × C) shows a significant effect on the wear loss.

#### 3.1.3. SWPC10T50 (Composite-C3)

Analysis of variance for means in Table 14 clearly indicates that sliding distance (P = 55.72%), sliding speed (P = 15.89%), load (P = 10.60%), interactions of (B) sliding speed × (C) sliding distance (P = 5.31%), and speed × load (A × B) (P = 5.43%) have a significant effect on the wear loss. But, interfaces of (B × C) load × distance (P = 3.14%) have little impact on wear loss because their values are much lower than error (P = 3.91%), and hence they are ignored. From response Table 15 for means, it is noticed that sliding distance has a key influence on wear loss followed by sliding speed and load.

The composite C3 ANOVA data are shown in Table 16. It is evident from the table that the sliding distance (P = 48.58%), sliding speed (P = 17.99%) and load (P = 12.60%), and the interaction between (A × C) speed and sliding distance (P = 10.75%) have a significant impact on wear loss, making them practically and analytically remarkable, though the interaction among (B × C) load and sliding distance (P = 3.66%) has less effect on the wear loss. From response Table 17 for S/N ratio of composite C3, it is shown that the control parameter (C) sliding distance, accompanied by (A) sliding speed and (B) load, has a significant impact on wear loss. The wear loss will be more due to increases in temperature at the interface.

**Figure 12:**
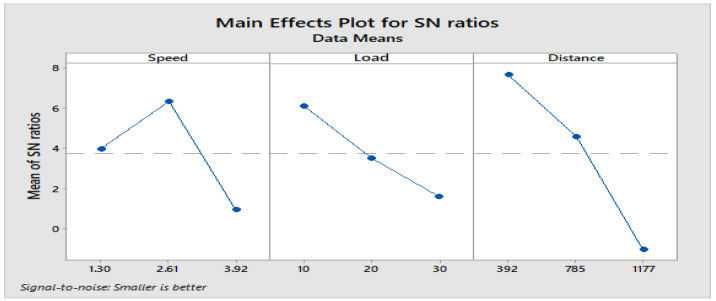
Main effects plot for S/N ratios of Composite-C3.

**Figure 13:**
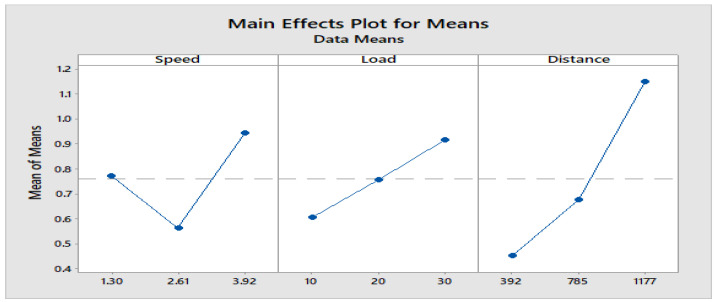
Main effects plot for means of Composite-C3.

**Figure 14:**
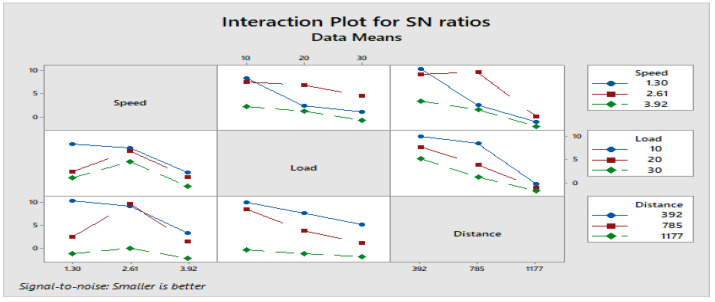
Interaction plot for S/N ratios of Composite-C3.

**Figure 15:**
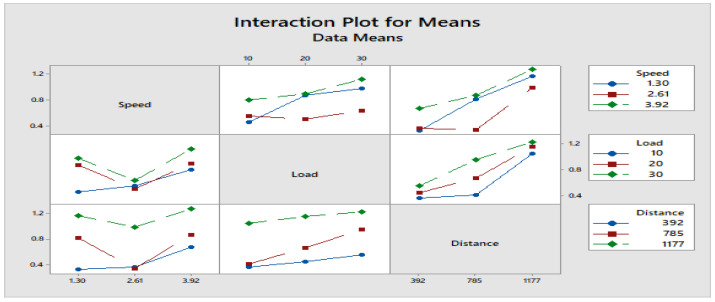
Interaction plot for means of Composite-C3.

**Figure 16:**
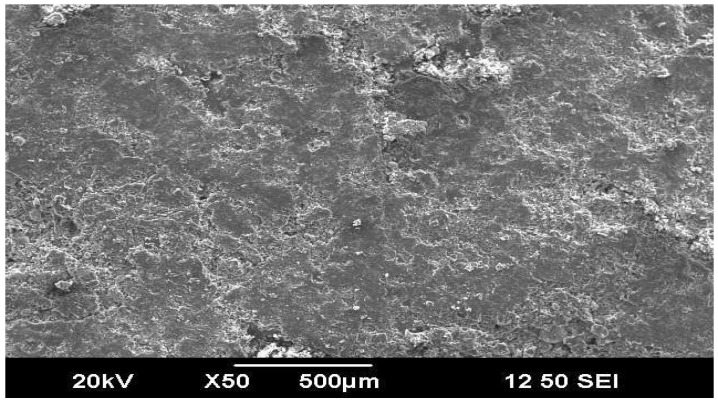
SEM images 10TSWPC30.

**Figure 17:**
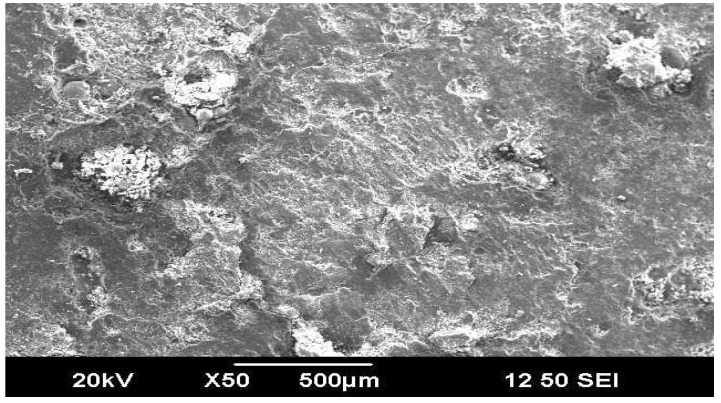
SEM images 10TSWC50.

The effects of factors on weight wear loss of composite specimens C3 are shown (Figure 12). The factors A3, B3, and C3 contribute to minor wear rate (Figure 13). In Figure 14 and Figure 15, the interaction graphs are illustrated. These figures demonstrate that the interaction (B × C) has a significant impact on wear loss. The wear weight loss of composite C3 will increase as the percentage of inclusion of wood flour increases to 50%. The bonding between resin and wood flour material is poor; hence, when this material comes in contact with a rotating disc, more material will be eroded from the surface when the sliding distance and speed increase as shown in Figure 16 and Figure 17.

#### 3.1.4. Confirmation Test

To confirm the outcomes and predict the ideal performance at the chosen values of significant parameters like A1, B1, and C2, the confirmation test is carried out. The most optimal set of combinations of parameters was found. Table 18 shows optimal process parameters for the wear test. The expected mean (Em) of the response characteristics can be stated as

(1) $$E_{m}=\left(A1-T\right)+\left(B1-T\right)+\left(C2-T\right)+T$$

where *T* = Average of S/N ratio

(2) $$E_{m}=\left(8.9-6.87\right)+\left(9.5-6.87\right)+\left(9.30-6.87\right)+6.87=13.96\;\mathrm{dB}$$

Table 19 shows a comparison between the experimental and estimated results of the wear test. The estimated mean of the wear loss was determined to be between 10.5 dB ≤ wear loss ≥ 15.6 dB and at the 95% confidence level [20]. It indicates the close agreement between estimated and experimental results.

## 4. Conclusions

The fabrication of PF-based wood–plastic composite consisting of saguvani wood particles as reinforcement material filled with coconut shell powder is technically feasible. The Taguchi method is used successfully to analyze the wear behavior of PF-based wood–plastic composite with sliding speed, load, and sliding distance as control parameters under dry sliding conditions. Including coconut shell powder as secondary particles in the PF-based wood–plastic composite improves the wear resistance of the composite. There is an appreciable reduction in wear loss of composite C1 with a 30% addition of wood flour. As the % of inclusion of wood powder increases, wear will increase. The poor bonding leads to more wear weight loss of composite C3 compared to other composite specimens C1 and C2 as the percentage of inclusion of wood flour increases to 50%. The experimental work reveals that sliding distance comparatively loads and sliding speed significantly influences the wear loss of these fabricated composite specimens C1, C2, and C3.

## Figures and Tables

**Table 1:** Density and grain fineness number of reinforcement materials.

Material (850 µm)	Density (kg/m³)	GFN
Saguvani wood flour	710	42.84
Coconut shell powder	1650	53.84

**Table 2:** Formulation of saguvani wood polymer composites (SWPC) [16].

Composite	PF (%)	WF (%)	CSP (%)
SWPC-10-T-30	70	25	5
SWPC-10-T-40	60	35	5
SWPC-10-T-50	50	45	5

**Table 3:** Grade and brand of all chemicals used.

Name of Chemical	Grade	Brand	Specifications
Sodium Hydroxide	S0033 (Mol.Wt.: 40.0)	Analisis	Assay: 96%
			Chloride: 0.1%
			Sulphate: 0.01%
Benzyl chloride	185558	Sigma-Aldrich	Assay: 99 %
			Form: Liquid
			Boiling Point: 177–181 °C
Acrylic acid	C_3_H_4_O_2_ = 72.06	Nice Chemicals	Assay: 99%
			Weight per ml at 200 °C: 1.048–1.050 g
			Refractive Index at 200 °C: 1.424
			Water: 0.2%

**Table 4:** Levels of Variables Used in the Experiments.

Levels	Units	1	2	3
Sliding speed	m/s	1.30	2.61	3.92
Sliding load	N	10	20	30
Sliding distance	m	392	785	1177

**Table 5:** Orthogonal Array L27 of Taguchi.

L27	1	2	3	4	5	6	7	8	9	10	11	12	13
1	1	1	1	1	1	1	1	1	1	1	1	1	1
2	1	1	1	1	2	2	2	2	2	2	2	2	2
3	1	1	1	1	3	3	3	3	3	3	3	3	3
4	1	2	2	2	1	1	1	2	2	2	3	3	3
5	1	2	2	2	2	2	2	3	3	3	1	1	1
6	1	2	2	2	3	3	3	1	1	1	2	2	2
7	1	3	3	3	1	1	1	3	3	3	2	2	2
8	1	3	3	3	2	2	2	1	1	1	3	3	3
9	1	3	3	3	3	3	3	2	2	2	1	1	1
10	2	1	2	3	1	2	3	1	2	3	1	2	3
11	2	1	2	3	2	3	1	2	3	1	2	3	1
12	2	1	2	3	3	1	2	3	1	2	3	1	2
13	2	2	3	1	1	2	3	2	3	1	3	1	2
14	2	2	3	1	2	3	1	3	1	2	1	2	3
15	2	2	3	1	3	1	2	1	2	3	2	3	1
16	2	3	1	2	1	2	3	3	2	1	2	3	1
17	2	3	1	2	2	3	1	1	2	3	3	1	2
18	2	3	1	2	3	1	2	2	3	1	1	2	3
19	3	1	3	2	1	3	2	1	3	2	1	3	2
20	3	1	3	2	2	1	3	2	1	3	2	1	3
21	3	1	3	2	3	2	1	3	2	1	3	2	1
22	3	2	1	3	1	3	2	2	1	3	3	2	1
23	3	2	1	3	2	1	3	3	2	1	1	3	2
24	3	2	1	3	3	2	1	1	3	2	2	1	3
25	3	3	2	1	1	3	2	3	2	1	2	1	3
26	3	3	2	1	2	1	3	1	3	2	3	2	1
27	3	3	2	1	3	2	1	2	1	3	1	3	2

**Table 6:** ANOVA for means of Composite-C1.

Source	DF	Seq SS	Adj SS	Adj MS	F	P	P %
Speed	2	0.14376	0.14376	0.07188	22.15	0.001	3.98
Load	2	0.35481	0.35481	0.17740	54.66	0	9.81
Distance	2	1.16803	1.16803	0.58402	179.95	0	32.30
Speed ∗ Load	4	0.04548	0.04548	0.01137	3.50	0.062	1.26
Speed ∗ Distance	4	0.05326	0.05326	0.01332	4.10	0.043	1.47
Load ∗ Distance	4	0.01668	0.01668	0.00417	1.29	0.352	0.46
Residual Error	8	0.02596	0.02596	0.00325			0.72
Total	26	1.80799					100

**Table 7:** Response table for means of Composite-C1.

Level	Speed	Load	Distance
1	0.4200	0.3922	0.3778
2	0.5444	0.4956	0.3667
3	0.5933	0.6700	0.8133
Delta	0.1733	0.2778	0.4467
Rank	3	2	1

**Table 8:** ANOVA for S/N ratios of Composite-C1.

Source	DF	Seq SS	Adj SS	Adj MS	F	P	P %
Speed	2	72.046	72.046	36.023	17.44	0.001	12.04
Load	2	128.803	128.803	64.401	31.17	0	21.53
Distance	2	322.77	322.77	161.385	78.12	0	53.96
Speed ∗ Load	4	20.081	20.081	5.02	2.43	0.133	3.36
Speed ∗ Distance	4	32.151	32.151	8.038	3.89	0.048	5.37
Load ∗ Distance	4	5.78	5.78	1.445	0.7	0.614	0.97
Residual Error	8	16.528	16.528	2.066			2.76
Total	26	1.80799					100

**Table 9:** Response table for S/N ratios of Composite-C1.

Level	Speed	Load	Distance
1	8.923	9.481	9.285
2	6.762	6.996	9.346
3	4.927	4.136	1.981
Delta	3.997	5.346	7.365
Rank	3	2	1

**Table 10:** ANOVA for means of Composite-C2.

Source	DF	Seq SS	Adj SS	Adj MS	F	P	P %
Speed	2	0.56272	0.56272	0.28136	16.56	0.001	16.82
Load	2	0.34259	0.34259	0.17129	10.08	0.007	10.24
Distance	2	1.92987	1.92987	0.96494	56.78	0.000	57.67
Speed ∗ Load	4	0.10337	0.10337	0.02584	1.52	0.284	3.09
Speed ∗ Distance	4	0.22228	0.22228	0.05557	3.27	0.072	6.64
Load ∗ Distance	4	0.04941	0.04941	0.01235	0.73	0.598	1.48
Residual Error	8	0.13596	0.13596	0.017			4.06
Total	26	3.34621					100.00

**Table 11:** Response table for means of Composite-C2.

Level	Speed	Load	Distance
1	0.63	0.5144	0.3944
2	0.4811	0.64	0.5322
3	0.8333	0.79	1.0178
Delta	0.3522	0.2756	0.6233
Rank	2	3	1

**Table 12:** ANOVA for S/N ratios of Composite-C2.

Source	DF	Seq SS	Adj SS	Adj MS	F	P	P %
Speed	2	139.49	139.49	69.745	43.44	0.000	19.97
Load	2	80.77	80.77	40.383	25.15	0.000	11.57
Distance	2	366.58	366.58	183.29	114.15	0.000	52.49
Speed ∗ Load	4	36.97	36.97	9.243	5.76	0.018	5.29
Speed ∗ Distance	4	51.21	51.21	12.802	7.97	0.007	7.33
Load ∗ Distance	4	10.48	10.48	2.62	1.63	0.257	1.50
Residual Error	8	12.85	12.85	1.606			1.84
Total	26	698.34					100.00

**Table 13:** Response table for S/N ratios of Composite-C2.

Level	Speed	Load	Distance
1	6.06062	7.29404	8.67352
2	7.37921	5.12324	6.73234
3	2.03545	3.058	0.06942
Delta	5.34376	4.23604	8.6041
Rank	2	3	1

**Table 14:** ANOVA for means of Composite-C3.

Source	DF	Seq SS	Adj SS	Adj MS	F	P	P %
Speed	2	0.648	0.648	0.32401	16.27	0.002	15.89
Load	2	0.4325	0.4325	0.21623	10.86	0.005	10.60
Distance	2	2.2729	2.2729	1.13643	57.05	0.000	55.72
Speed ∗ Load	4	0.2215	0.2215	0.05538	2.78	0.102	5.43
Speed ∗ Distance	4	0.2167	0.2167	0.05418	2.72	0.107	5.31
Load ∗ Distance	4	0.1279	0.1279	0.03197	1.60	0.263	3.14
Residual Error	8	0.1594	0.1594	0.01992		3.91	
Total	26	4.0788					100.00

**Table 15:** Response table for means of Composite-C3.

Level	Speed	Load	Distance
1	0.7722	0.6056	0.4522
2	0.5644	0.7589	0.6789
3	0.9433	0.9156	1.1489
Delta	0.3789	0.3100	0.6967
Rank	2	3	1

**Table 16:** ANOVA for S/N ratios of Composite-C3.

Source	DF	Seq SS	Adj SS	Adj MS	F	P	P %
Speed	2	130.95	130.95	65.477	31.83	0.000	17.99
Load	2	91.70	91.70	45.848	22.29	0.001	12.60
Distance	2	353.67	353.67	176.836	85.96	0.000	48.58
Speed ∗ Load	4	30.36	30.36	7.59	3.69	0.055	4.17
Speed ∗ Distance	4	78.28	78.28	19.571	9.51	0.004	10.75
Load ∗ Distance	4	26.64	26.64	6.659	3.24	0.074	3.66
Residual Error	8	16.46	16.46	2.057		2.26	
Total	26	728.06		100.00			

**Table 17:** Response table for S/N ratios of Composite-C3.

Level	Speed	Load	Distance
1	3.9716	6.1214	7.7076
2	6.3438	3.5312	4.6053
3	0.9619	1.6246	−1.0357
Delta	5.3819	4.4968	8.7433
Rank	2	3	1

**Table 18:** The optimal set of parameters for the wear test.

Parameters	Symbols	Composites	Optimum Settings
Speed in (m/s)	A	C1	1.30
		C2	1.30
		C3	1.30
Load in (*N*)	B	C1	10
		C2	10
		C3	10
Sliding distance in (m)	C	C1	785
		C2	392
		C3	392

**Table 19:** Comparison between experimental and estimated results of the wear test.

Optimum Level				
	**Composites**	**Estimation**	**Experimental**	**Deviation**
Level	C1	A1B1C2	A1B1C2	-
	C2	A1B1C1	A1B1C1	-
	C3	A1B1C1	A1B1C1	-
Wear	C1	1.19	0.18	1.01
	C2	1.38	0.26	1.29
	C3	1.62	0.30	1.32
S/N ratio	C1	13.96	14.49	0.53
	C2	15.25	11.70	3.55
	C3	13.87	10.45	3.41

## Data Availability

Not applicable.
